# Supplementary material for: Long term prophylactic anticoagulation for portal vein thrombosis after splenectomy: A systematic review and meta-analysis
Source: PLoS One. 2023 Aug 15;18(8):e0290164. doi: 10.1371/journal.pone.0290164 (PMC10426921; doi:10.1371/journal.pone.0290164)
Supplement: S1 File — (DOCX) [file pone.0290164.s001.docx]

1.PVST incidence

input the data

metan te tn ce cn, label(namevar=author, yearvar=year) by(time) fixed or boxsca(10)

2.bleeding incidence

input the data

metan te tn ce cn, label(namevar=author, yearvar=year) fixed or boxsca(10)

3.funnel plot of PVST

input the data

metan te tn ce cn, label(namevar=author, yearvar=year) by(time) fixed or boxsca(10)

gen logor=log(_ES)

gen selogor=_selogES

metafunnel logor selogor

3.egger test of PVST

input the data

metan te tn ce cn, label(namevar=author, yearvar=year) by(time) fixed or boxsca(10)

gen logor=log(_ES)

gen selogor=_selogES

metabias logor selogor, graph(egger)

4.trim and fill

input the data

metan te tn ce cn, label(namevar=author, yearvar=year) by(time) fixed or boxsca(10)

gen logor=log(_ES)

gen selogor=_selogES

metatrim logor selogor, funnel

5.funnel plot of bleeding

input the data

metan te tn ce cn, label(namevar=author, yearvar=year) fixed or boxsca(10)

gen logor=log(_ES)

gen selogor=_selogES

metafunnel logor selogor
