# Supplementary material for: Long term prophylactic anticoagulation for portal vein thrombosis after splenectomy: A systematic review and meta-analysis
Source: PLoS One. 2023 Aug 15;18(8):e0290164. doi: 10.1371/journal.pone.0290164 (PMC10426921; doi:10.1371/journal.pone.0290164)
Supplement: Attachment — Submitted filename: Review Report (PONE-D-23-13885).docx [file pone.0290164.s002.docx]

**Review Report: Ref. No.:  PONE-D-23-13885**

In this study, the authors aimed to assess the effectiveness and safety of anticoagulants in preventing portal vein system thrombosis (PVST) in patients with cirrhosis following splenectomy. Additionally, the study aimed to determine the optimal timing for administering anticoagulants. To achieve this, a comprehensive search of literature databases including PubMed, Embase, and China Biology Medicine disc (CBM) was conducted. The search aimed to identify relevant studies that compared the outcomes of cirrhotic post-splenectomy patients who received anticoagulants to those who did not. The parameters analyzed encompassed the occurrence of PVST and postoperative bleeding.

The topic is interesting and falls under the scope of the journal. The article is well organized and well structured. However, the authors need to address the following comments.

**Major Comments:**

1. Provide the syntax codes of STATA 12.0 in the appendix.
2. Revise section 2.5 and interpret different statistical tools used in this study for the convenience of the readers such as confidence interval, Chi-square test, Egger’s test, etc.

**Minor comments:**

1. The title is too long for the reader; it should be precise.
2. Table 1 font is too small; readjust to make it more visible.
3. Page 2, line 15, write as “explore the optimal time of anticoagulant administration”.
4. Page 10, line 40, write, “Cirrhosis can increase portal vein pressure and lead to hypersplenism”.
5. The two investigators examined the full text, tables, and figures of relevant literature to extract data from articles that had been included in this study.
6. There are some grammatical mistakes in the article please revise the article thoroughly and remove all the errors.
7. Conclusions are not clearly specified.
8. Some of the references need revision such as reference 7 and 19.
