# Supplementary material for: Long term prophylactic anticoagulation for portal vein thrombosis after splenectomy: A systematic review and meta-analysis
Source: PLoS One. 2023 Aug 15;18(8):e0290164. doi: 10.1371/journal.pone.0290164 (PMC10426921; doi:10.1371/journal.pone.0290164)
Supplement: Attachment — Submitted filename: Response to Reviewers.docx [file pone.0290164.s003.docx]

Dear reviewers,

Thank you very much for your comments and professional advice. Based on your suggestion and request we have made corrected modifications on the revised manuscript. We hope that our work can be improved again. The details as follows:

**Reviewer #1**

Major Comments:

1. Provide the syntax codes of STATA 12.0 in the appendix.

**The author’s answer**: We uploaded my syntax code of Stata 12.0 as supporting information.

2. Revise section 2.5 and interpret different statistical tools used in this study for the convenience of the readers such as confidence interval, Chi-square test, Egger’s test, etc.

**The author’s answer**: We have revised the section in the article. The details as follows: “The meta‑analysis was performed using stata12.0 .The incidences of postoperative PVST and postoperative bleeding were treated as dichotomous data and pooled odds ratios (OR) with 95% confidence interval (CI) were used to perform analysis. The OR is used to reflect differences in exposure between experiments and controls, thus establishing a link between disease and exposure factors. P<0.05 was considered to indicate a statistically significant difference between the two groups. In terms of the heterogeneity test, when the statistics P > 0.05, I2 < 50%, it can be assumed that there is no significant statistical difference in the included data, so the fixed effect model is adopted. When the statistics P ≤ 0.05 and I2 ≥ 50%, indicating that there are significant statistical differences among the included data. The random effect model is adopted. A funnel plot and Egger’s test were designed to establish the existence of publication bias. The trim-and-fill method was performed to further assess the potential publication bias. ”

Minor comments:

1. The title is too long for the reader; it should be precise.

**The author’s answer**: We have streamlined the title. The details as follows: “Long term prophylactic anticoagulation for portal vein thrombosis after splenectomy: A systematic review and meta-analysis”.

2. Table 1 font is too small; readjust to make it more visible.

**The author’s answer**: We have enlarged the font in the table.

3. Page 2, line 15, write as “explore the optimal time of anticoagulant administration”.

**The author’s answer**: We have revised the sentence. The details as follows: “The aim of this study was to evaluate the efficacy and safety of the anticoagulants for the prevention of portal vein system thrombosis (PVST) in patients with cirrhosis after splenectomy and explore the optimal time of anticoagulant administration.”

4. Page 10, line 40, write, “Cirrhosis can increase portal vein pressure and lead to hypersplenism”.

**The author’s answer**: We have revised the sentence. The details as follows: “Cirrhosis can increase portal vein pressure and lead to hypersplenism, and splenectomy is often used to treat patients with portal hypertension and hypersplenism.”

5. The two investigators examined the full text, tables, and figures of relevant literature to extract data from articles that had been included in this study.

**The author’s answer:** We have revised the sentence. The details as follows: “The two investigators examined the full text, tables and figures of relevant literature to extract data from articles that have been included in this study. ”

6. There are some grammatical mistakes in the article please revise the article thoroughly and remove all the errors.

**The author’s answer:** We have checked the article and fixed some grammatical mistakes.

7. Conclusions are not clearly specified.

**The author’s answer:** We have revised the conclusions. The details as follows: “Our meta-analysis suggested that combined anticoagulation with LMWH and warfarin can decrease the incidence of PVST in post-splenectomy cirrhotic patients without an increased risk of bleeding. Patients after splenectomy can have short term injections of LMWH followed by long term use of warfarin. And patients were best served with warfarin for 6 months after splenectomy to prevent portal vein thrombosis.”

8. Some of the references need revision such as reference 7 and 19.

**The author’s answer:** We have revised the references according to the requirements of plos one.

Thank you for your attention and time.

Yours sincerely,

Zheng Liao

Corresponding author:

Name: Jinlong Liu

E-mail: liujl800813@163.com
